# Modulating the Microbiome and Immune Responses Using Whole Plant Fibre in Synbiotic Combination with Fibre-Digesting Probiotic Attenuates Chronic Colonic Inflammation in Spontaneous Colitic Mice Model of IBD

**DOI:** 10.3390/nu12082380

**Published:** 2020-08-09

**Authors:** Tanvi Shinde, Ravichandra Vemuri, Sonia Shastri, Agampodi Promoda Perera, Shakuntla V. Gondalia, David J. Beale, Avinash V. Karpe, Rajaraman Eri, Roger Stanley

**Affiliations:** 1 Centre for Food Innovation, Tasmanian Institute of Agriculture, University of Tasmania, Launceston, Tasmania 7250, Australia; 2 Gut Health Research Group, School of Health Sciences, College of Health and Medicine, University of Tasmania, Launceston, Tasmania 7250, Australia; rvemuri@wakehealth.edu (R.V.); sonia.shastri@utas.edu.au (S.S.); agampodi.perera@utas.edu.au (A.P.P.); rajaraman.eri@utas.edu.au (R.E.); 3 Department of Pathology, Wake Forest School of Medicine, Medical Center Boulevard, Winston-Salem, NC 27157, USA; 4 Centre for Human Psychopharmacology, Swinburne University of Technology, Hawthorn, Victoria 3122, Australia; Shakuntla.Gondalia@csiro.au; 5 Health and Biosecurity, Commonwealth Scientific and Industrial Research Organization (CSIRO), Gate 13 Kintore Avenue, South Australia 5000, Australia; 6 Land and Water, Commonwealth Scientific and Industrial Research Organization (CSIRO), Ecosciences Precinct, Dutton Park, Queensland 4102, Australia; david.beale@csiro.au (D.J.B.); avinash.karpe@csiro.au (A.V.K.)

**Keywords:** synbiotic, prebiotic, probiotic, Inflammatory Bowel Diseases (IBD), *Bacillus* spores, whole plant sugarcane fibre, Short chain fatty acids (SCFAs), dysbiosis, gut microbiota

## Abstract

A probiotic and prebiotic food ingredient combination was tested for synergistic functioning in modulation of the colonic microbiome and remediation of the gastrointestinal immune and inflammatory responses in a spontaneous colitic mouse model. *Bacillus coagulans* MTCC5856 spores with capability to metabolise complex plant polysaccharides were supplemented with complex whole-plant prebiotic sugarcane fibre (PSCF). The combined and individual efficacies were tested for their influence on the outcomes of chronic inflammation in *Muc2* mutant colitic Winnie mice. The mice were fed normal chow diet supplemented with either ingredient or a combination for 21 days. Synbiotic combined supplementation ameliorated clinical symptoms and histological colonic damage scores more effectively than either *B. coagulans* or PSCF alone. PSCF and *B. coagulans* alone also induced considerable immunomodulatory effects. Synbiotic supplementation however was the most efficacious in modulating the overall immune profile compared to the unsupplemented Winnie-control. The augmented synbiotic effect could potentially be due to a combination of increased levels of fermentation products, direct immune-modulating abilities of the components, their capability to reduce colonic epithelial damage and/or modulation of the microbiota. The beneficial effects of the supplementation with a complex plant fibre and a fibre-degrading probiotic parallel the effects seen in human microbiota with high plant fibre diets.

## 1. Introduction

The role of the gastrointestinal microbiome in promoting and maintaining good health is being increasingly recognised [1]. Westernised dietary choices of energy rich, lower nutrient density foods are thought to be a contributing factor to rising incidences of inflammatory bowel diseases (IBD) [2]. IBD, including Ulcerative colitis (UC) and Crohn’s disease (CD) are characterised by the chronic inflammatory conditions of the gastrointestinal tract. A three part pathophysiological circuit involving aberrant immune response, dysbiotic intestinal microbiota (and the associated metabolic pathways) and aberrant intestinal barrier function have been considered leading factors for causing ongoing chronic inflammation [3]. The ability to counter such dysfunctions is focused on better dietary choices of increased dietary fibres (DF) from fruit and vegetable consumption [4]. DF can influence the composition of gut microbiota and the associated bacterial metabolic pathways, as well as interact with the immune system [5]. However, achieving population health through recommended daily intake of fruit and vegetables has been problematic [6]. Mechanistic understanding of the links between diet, microbiome and health is being sought to focus food development towards heathier outcomes. Dietary diversity has been linked with health outcomes [7]. Dietary strategies for relief of gastrointestinal symptoms, such as using purified plant fibres or avoiding plant fibre with fermentable saccharides and polyols (FODMAPS) may be counterproductive to microbial diversity and long-term health [8,9]. Increased consumption of fruit and vegetables bred for high yields of carbohydrates and oils may also be counterproductive with energy loads from these concentrated sources limiting the intake of complex fibres that could promote microbiome health. Sugarcane is a grass bred for high sucrose yields. Conventionally, it is finely ground for high extraction yield with the resulting bagasse fibre being depleted in complex polysaccharides as well as being damaged in high temperature drying for energy recovery through steam generation. A minimally processed sugarcane flour is produced with water extraction to remove the sugar and subsequent drying and grinding to a flour that retains the structural features of plant cellular materials [10,11]. It consequentially has a complex plant cell wall composition in a palatable form without the associated levels of digestible carbohydrates of fruit and vegetables [12]. It can therefore be used as a food ingredient to manipulate levels of complex plant cellular material in the diet.

The success of the complex dietary fibre (DF) strategy, however, is largely governed by the gut microbial diversity and complexity. The production of short-chain fatty acids (SCFAs), via microbial fermentation of DF in the gut, is a major potential mechanistic mediator to host physiology and immune homeostasis [2,13,14]. However, perturbations in the composition of the gut microbiota and its metabolic capacity associated with IBD compromises its effectiveness. Particularly, depletion of fibre-degrading-SCFA-producing bacterial members and concurrently reduced SCFA levels are implicated in gut inflammatory disorders [2,14]. Therefore, administration of fibre alone might not be effective in generating potent SCFA levels that are known to confer anti-inflammatory effects [14]. Thus, there is a critical challenge in harnessing health outcomes through consumption of fibre-rich diet during inflammatory dysbiotic state due to lack of fibre-digesting bacterial members. Similarly, replenishing the beneficial bacteria through probiotic ingestion alone without a fibre substrate would also be unreliable to affect health outcomes. Thus, co-administration of probiotic bacteria that can synergistically metabolise the administered complex prebiotic DF as synbiotic and consequently improve the probiotic activity to produce beneficial metabolites and influence the microbiota is a judicious approach in positively modulating the microbial environment to induce immune homeostasis.

The use of various bacterial probiotics, selected to show at least transient impact on gut health when consumed at high enough levels, is another strategy to manipulate the microbiome. However, the ability of empiric probiotic supplements in relatively low dosages to impact the natural microbiome is questionable [15,16]. The gastrointestinal (GI) tract microbiome is regulated by complex mechanisms, but a relatively stable composition is usually generated with different families of microbes exploiting niches in the available digesta and microbial metabolites. Conventionally, probiotic development does not seek to influence the microbial ecology of the gut except at the mucosal interface where interaction with intestinal sensing systems, and microbial exposure is controlled by a complex immune regulating system at the mucosal barrier [17]. *Bacillus coagulans* however is a lactic acid bacterium. Various strains of it are used in thermophilic industrial fermentations of foods, plant saccharides and agricultural wastes to primarily produce principally lactic acid [18,19,20]. *B. coagulans* can metabolise a variety of plant substrates rich in insoluble cell wall components. *B. coagulans* MTCC5856 probiotic spores are reported as being optimally grown on complex plant fibres and are confirmed to metabolise complex plant materials to generate lactic acid and SCFAs [21,22,23]. Cellulolytic degradation ability, however, is highly limited in the human gut but greatest in individuals having methanogens in their gut microbiota [24]. *B. coagulans* MTCC5856 spores are also generally regarded as safe (GRAS) affirmed and have been demonstrated to confer substantial immunomodulatory, anti-inflammatory and anti-diarrhoeal effects [25,26,27]. However, a catalytic role in positively altering the microbial composition and physiology of the dysbiotic GI tract through accelerating digestion of plant cellular material and producing bacterial regulators like lactic acid and SCFAs has not been explored. A catalytic mechanism, while temporary, as *B. coagulans* does not establish permanently in the human gut, offers a mechanistic route to impact the occurrence and pathology of IBD.

Our previous study [27], clearly demonstrated that pre-conditioning of mice with synbiotic combination of prebiotic sugarcane fibre (PSCF) and *B. coagulans* spores in exerting excellent preventive effect in chemically-induced acute colitic mice. However, the therapeutic efficacy of this synbiotic treatment in mitigating established chronic gut inflammation is not known. This study therefore aimed to investigate the therapeutic efficacy of the whole-plant PSCF and *B. coagulans*, alone and in combination as synbiotic, in the spontaneous chronic-colitic-Winnie (*Muc2* mutant) mouse model of IBD [28,29] and further examine its underlying mechanisms.

## 2. Materials and Methods

### 2.1. Probiotic Bacteria and Prebiotic Dietary Fibre

LactoSpore^®^ (Sabinsa Corporation, East Windsor, NJ, USA) containing the probiotic strain *Bacillus coagulans* MTCC 5856 (6 × 10^9^ spores/g) was produced by Sami Labs Limited (Bangalore, India) and supplied by Sabinsa Corporation (Australia). Kfibre™, whole plant prebiotic sugarcane fibre (PSCF) was supplied by KFSU Pty Ltd., Queensland, Australia. The nutritional composition of Kfibre is detailed in Supplementary Table S1.

### 2.2. Animals

Thirty-two, six-week-old Winnie mice (homozygous *Muc2* mutant; C57BL/6J background) of both sexes were obtained from the University of Tasmania animal breeding facility and housed in a temperature-controlled environment with a 12-h day/night light cycle. Individual body weights were assessed daily after an initial acclimation period of 7 days. All mice had access to radiation-sterilised rodent feed pellets (Barastoc Rat and Mouse, Ridley AgProducts, Harristown, Australia) and autoclaved tap water for drinking ad libitum during experiments. The nutritional composition of rodent chow is detailed in Supplementary Table S2. All animal experiments were approved by the Animal Ethics Committee of the University of Tasmania (ethics approval number: A0015840) and conducted in accordance with the Australian Code of Practice for Care and Use of Animals for Scientific Purposes (8th Edition 2013). All efforts were made to minimize animals’ suffering and to reduce the number of animals used.

### 2.3. Study Design and Treatments

**Figure 1:**
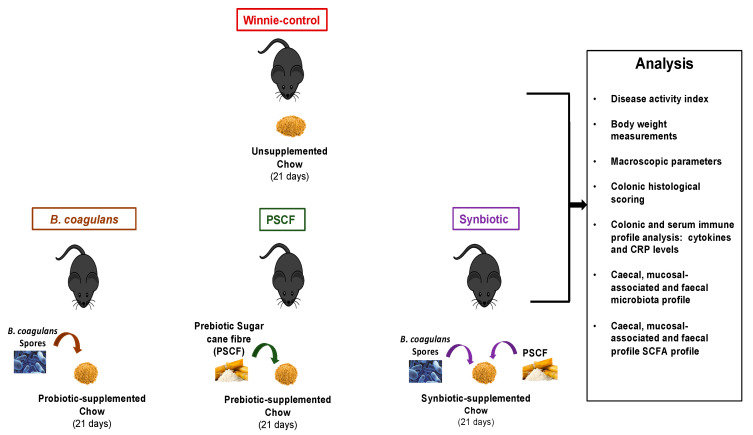
Experimental design of in-vivo feeding trial to analyse therapeutic efficacy of *B. coagulans* spores, prebiotic sugarcane fibre (PSCF) and Synbiotic in chronic-colitic-Winnie mice. Colitic Winnie mice (*n* = 8 per group) that received normal drinking water were fed chow supplemented with either *B. coagulans* spores, PSCF or their Synbiotic combination for 21 days.

Following 1 week of acclimation, Winnie mice at 7 weeks of age were randomly allocated into the following 4 groups (*n* = 8 per group): (1) Winnie-control, (2) Probiotic *B. coagulans* MTCC 5856 (*B. coagulans*), (3) Whole plant prebiotic sugarcane fibre (PSCF) and (4) Synbiotic supplement. The experimental design of the mice feeding trial is illustrated in Figure 1. Mice in the Winnie-control group received 4 g chow mash (standard chow pellet blended with water). The *B. coagulans* group received 4 g chow mash supplemented with probiotic *B. coagulans* MTCC 5856 spores (2 × 10^9^ CFU/day/mouse). The PSCF group received 4 g chow mash supplemented with Kfibre™ (200 mg/day/mouse). Synbiotic mice received 4 g chow, each supplemented with *B. coagulans* MTCC 5856 spores and Kfibre. The chow mash was prepared fresh each day. The mice were single-caged throughout the experiment to measure the defined daily intake of respective treatments from prepared chow mash. The mice were fed these treatments for 21 days. There were no differences in the daily intake of the treatment-supplemented chow among groups (data not shown). Mice were sacrificed on day 22 by CO_2_ asphyxiation.

### 2.4. Clinical Scoring and Histological Analysis

Body weight and disease activity index (DAI) were determined daily in all Winnie and graded as detailed previously [27,30]. DAI was determined by combining the scores from stool consistency, hemoccult reactivity and presence of blood in the stool. Briefly, the following parameters were used for calculation of DAI: (a) stool consistency (score 0 = normal, score 1 = soft but still formed, score 2 = very soft/loose stool, score 3 = diarrhoea/watery stool); and (b) blood in stool (score 0 = negative hemoccult, score 1 = positive hemoccult, score 2 = Blood traces in stool visible, score 3 = rectal bleeding). Body weights were calculated for each animal throughout the experiments and weight calculated as percent weight change to the weight immediately before administration of supplemented chow (day 0). Faecal samples from Winnie were collected at the end of the experiment (day 21) and stored at −80 °C for SCFAs and microbiota analyses.

After sacrificing the mice, the colons were excised from the caecum to the anus as described previously [27,30]. Spleen weight, colon length and colon weight were recorded for the measurement of macroscopic markers of inflammation. The colon was bisected longitudinally, and one half was prepared using the Swiss roll technique whereas the remaining colonic tissue was dissected out, segregated into proximal colon (PC) and distal colon (DC) and snap-frozen for molecular analyse as described previously [27]. Swiss rolls underwent 24 h fixation in 10% (*v/v*) neutral-buffered formalin. Swiss rolls were subsequently transferred to 70% ethanol prior to progressive dehydration, clearing and infiltration with HistoPrep paraffin wax (Fisher Scientific, Philadelphia, PA, USA). Swiss rolls were then embedded in wax and 5 μm sections were cut using a rotary microtome. For histological analysis, proximal and distal colon tissue sections (*n* = 8 per group) were stained with haematoxylin and eosin stain (H and E; HD Scientific, Sydney, Australia). Slides stained with H&E (*n* = 6 per group) were graded blindly for the severity of tissue damage at distal and proximal regions based on the previously described scoring system [30,31]. Briefly, frequency of inflammatory infiltrate graded 0–3, goblet cell loss graded 0–3, crypt architectural distortion graded 0–3, frequency of crypt abscess graded 0–3, crypt hyperplasia graded 0–3, muscle thickening (oedema) graded 0–3, ulceration graded 0–3. The histological inflammation score for each proximal and distal colon region was derived from the sum of the score for each aforementioned criterion. All images were captured on a Leica DM500 microscope using a Leica ICC50 W camera (Leica Microsystems, Wetzlar, Germany).

### 2.5. Tissue Explant Culture and Serum Cytokine Measurements

The cytokine levels in colon tissues (*n* = 3) and serum (*n* = 3) were determined by immunoassay using a Bio-Plex Pro Mouse cytokine 23-plex kit (#M60009RDPD, Bio-Rad Laboratories, Hercules, Australia) following the manufacturer’s instructions and concentrations analysed in duplicate using a Bio-Plex 200 instrument (Bio-Rad Laboratories) and Bioplex Manager software, version 6 (Bio-Rad Laboratories), respectively [27]. For tissues, the cytokine levels were normalized by dividing the cytokine results (pg/mL) by the measured biopsy weight (g), and the cytokines are presented as pg/g of tissue.

### 2.6. Serum C-Reactive Protein Analysis

The levels of C-reactive protein (CRP) in serum from respective groups (*n* = 3 samples/group) were analysed using Mouse C-Reactive Protein/CRP Quantikine Elisa kit (MCRP00, R and D Systems, Sydney, Australia) following the manufacturer’s instructions. The results were expressed as μg/mL.

### 2.7. Microbiota Analysis by 16 s rRNA High-Throughput Sequencing

The total DNA was extracted from caecal, mucosal-associated and faecal samples (*n* = 5 per group) of Winnie mice using the QIAamp DNA Stool Mini Kit (Qiagen, Melbourne, VIC, Australia). DNA extracted from C57BL/6J wild-type (WT) mice were also used for comparison. The samples underwent high-throughput sequencing on the Illumina MiSeq platform at the Australian Genome Research Facility (University of Queensland, Brisbane, QLD, Australia) according to the methods detailed earlier [32]. Polymerase chain reaction (PCR) amplicons spanning the 16 S rRNA V1–V3 hypervariable region with 27 F forward primer (5′-AGAGTTTGATCMTGGCTCAG-3′) and 519 R reverse primer (5′-GWATTACCGCGGCKGCTG-3′) were sequenced. Paired-end reads were assembled by aligning the forward and reverse reads using PEAR1 (version 0.9.5). Primers were identified and trimmed. Trimmed sequences were processed using Quantitative Insights into Microbial Ecology (QIIME 1.8) 4 USEARCH 2.3 (version 8.0.1623) and UPARSE software [33]. Using USEARCH tools, sequences were quality filtered; full-length duplicate sequences were removed and sorted by abundance. Singletons or unique reads in the data set were discarded. Sequences were clustered followed by chimera filtering using “rdp_gold” database as a reference [34]. To obtain several reads for each Operational Taxonomic Units (OTUs), reads were mapped back to OTUs with a minimum identity of 97%. Using QIIME, taxonomy was assigned using Greengenes database5 (Version 13_8, August 2013) [35]. Image analysis was performed in real time by the MiSeq Control Software (MCS) v2.6.2.1 and Real-Time Analysis (RTA) v1.18.54, running on the instrument computer. RTA performs real-time base calling on the MiSeq instrument computer. The Illumina bcl2fastq 2.20.0.422 pipeline was used to generate the sequence data [34,35]. 16 S rRNA gene sequences were analysed using MEGAN6 (Community edition version) [36], Microbiome analyst [37] and QIIME 2. Statistical analysis of Bray-Curtis dissimilarities was calculated using the relative abundances of bacterial genera using Adonis function in R (version 3.2).

### 2.8. Volatile SCFA Analysis

GC-MS analysis of 100–150 mg fresh weight (stored at −80 °C) of caecal, mucosal-associated and faecal samples (*n* = 5 per group) from Winnie and WT mice was conducted for volatile SCFA profiling following the method described previously [27,32]. Briefly, following sample preparation, the GC-MS analysis was performed on an Agilent 6890B GC oven coupled to a 5977B mass spectrometer (MS) detector (Agilent Technologies, Mulgrave, VIC, Australia) fitted with an MPS autosampler (Gerstel GmbH & Co.KG, Mülheim an der Ruhr, Germany). The GC oven was fitted with two 15 m HP-5MS columns (0.25 mm ID and 0.25 µm film thickness; 19091S-431 UI (Ultra Inert), Agilent Technologies, VIC, Australia) coupled to each other through a purged ultimate union (PUU) for the use of post-run backflushing. The sample (1.0 µL) was introduced via a multimode inlet (MMI) operated in split mode (1:20). The column was maintained at 40 °C for 5 min, followed by an increase to 250 °C at a rate of 10 °C/min. This was followed by a second increment to 310 °C at a rate of 60 °C/min. The column was held at 310 °C for 1 min. The mass spectrometer was kept in extractor ion mode (EI mode) at 70 eV. The GC-MS ion source temperature and transfer line were kept at 250 °C and 280 °C, respectively. Detector voltage was kept at 1054 V. The MS detector was turned off for the first 3 min and, at 4.0–4.8 min and 12.5–13.2 min time windows until the excess derivatization reagent (chloroformate/hexane solvents) were eluted from the column. This ensured that the source filament was not saturated and damaged. The scan range was kept in the range of *m*/*z* 35–350 (35–350 Daltons). Data acquisition and spectral analysis were performed as described in a previous study [35] and qualitative identification of metabolites was performed according to the Metabolomics Standard Initiative (MSI) chemical analysis workgroup [36] using standard GC-MS reference metabolite libraries (NIST 17), and an in-house CF-based metabolomics library developed after Smart et al. [37] with the use of Kovats retention indices based on a reference *n*-alkane standard (C8-C40 Alkanes Calibration Standard, Sigma-Aldrich, Castle Hill, NSW, Australia).

### 2.9. Statistical Analysis

All data are presented as means ± SEMs. The statistical analysis was performed using GraphPad Prism Software (Version 7.0) The data were evaluated using One-way analysis of variance (ANOVA) followed by Tukey’s post-hoc test to determine statistical differences between the groups against Winnie-control samples. For the analysis of DAI and body weight changes during the experimental period, Two-way ANOVA was used followed by Tukey’s post-hoc test, setting treatment and the time as the variables. A *p*-value of <0.05 was considered significant. To determine overall microbial variation in the five groups, a principal coordinate analysis (PCoA) was used with Bray-Curtis ecological indexing and Euclidean distances as the similarity measure and Ward’s linkage as a clustering algorithm [32]. Two bacterial alpha (α-) biodiversity indices were evaluated, i.e., the Inverse Simpson Index and the Shannon Index. for both indices, an increased value indicates greater diversity [38]. The data were evaluated with one-way analysis of variance (ANOVA) and using Tukey’s test for multiple comparisons with a statistical significance of *p* < 0.05. For comparative microbial analysis, a linear discriminant effect size (LEfSe) analysis was performed (α = 0.05), logarithmic Linear Discriminant Analysis (LDA) score threshold = 1.0.

## 3. Results

### 3.1. Dietary Synbiotic Supplementation Induced Considerable Attenuation of Clinical Manifestations

**Figure 2:**
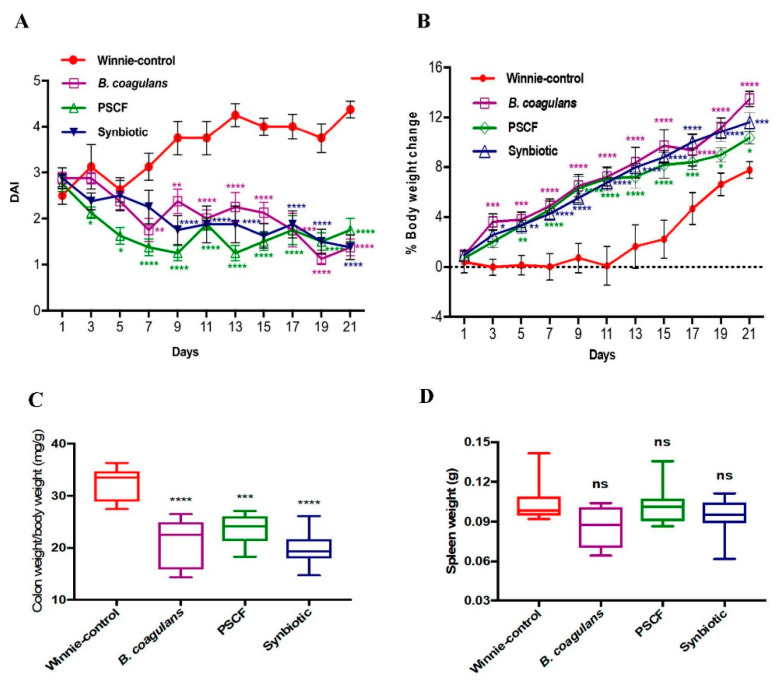

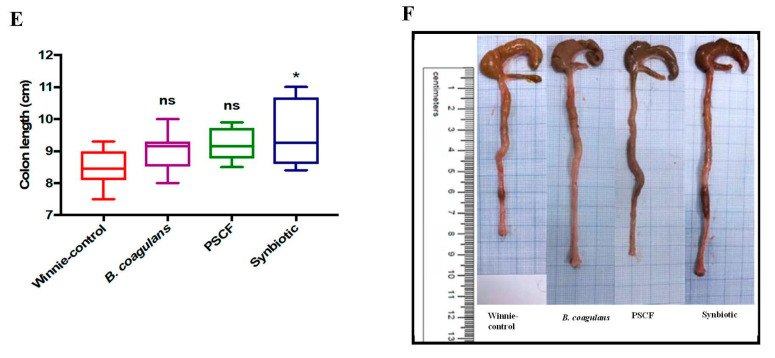
Effect of *B. coagulans* spores, PSCF and Synbiotic on clinical manifestations in chronic-colitic-Winnie mice. (**A**) Disease Activity Index (DAI), (**B**) % body weight change. Statistical significance among groups evaluated by two-way repeated-measures analysis of variance (ANOVA) * *p* < 0.05, ** *p* < 0.01, *** *p* < 0.001, **** *p*< 0.0001 vs. Winnie-control group and data expressed as mean ± SEM (*n* = 8 per group). Colon weight/body weight ratio (**C**), Spleen weight (**D**), Colon length (**E**) and macroscopic appearance of colon (**F**). Data expressed as mean ± SEM (*n* = 8 per group), evaluated by one-way ANOVA followed by Tukey’s Test. NS = non-significant, PSCF-Prebiotic sugarcane fibre.

DAI (stool consistency and blood in faeces) and body weight changes were evaluated to determine the efficacy of the treatments in reducing the severity of disease symptoms in colitic-Winnie (Figure 2). Compared with the Winnie-control group that showed severe colitis symptoms (loss of body weight and diarrheic/bloody faeces), supplementation with *B. coagulans*, PSCF and Synbiotic significantly reduced the DAI levels as well as prevented body weight loss throughout the experiment (Figure 2A). At the end of the experiment, DAI of Winnie-control group was significantly higher (*p* < 0.0001) compared with that of treated mice. *B. coagulans*, PSCF and Synbiotic lowered the DAI by 69%, 60% and 69%, respectively. Noticeably, PSCF was most effective in reducing DAI as early as day 3 mainly owing to improvement in stool consistency in comparison with *B. coagulans* and Synbiotic. In contrast to Winnie-control mice on day 21 (Figure 2B), mice supplemented with *Bacillus,* PSCF and Synbiotic treatments recovered body weight loss by 73.89%, 33.23% and 49.79%, respectively.

The macroscopic evaluation of colonic segments revealed the beneficial effects of all three supplementations used in the study. This is evidenced by marked reduction in colon weight/body weight ratio (*B. coagulans,* 21.01 ± 1.7; PSCF, 23.57 ± 1.0 and Synbiotic, 19.79 ± 1.2 mg/g) compared with Winnie-control group (32.29 ± 1.2 mg/g) (Figure 2C). None of the supplementations were effective in reducing the spleen enlargement (Figure 2D) that is also associated with colonic inflammation [39]. Synbiotic supplementation was also significantly effective in reducing the colon length shortening (9.5 ± 0.4) compared to the shorter colon length of Winnie-control mice (8.4 ± 0.2 cm) (Figure 2E,F).

### 3.2. Synbiotic Supplementation Reduced Histological Alterations in Chronic-Colitic-Winnie Mice

**Figure 3:**
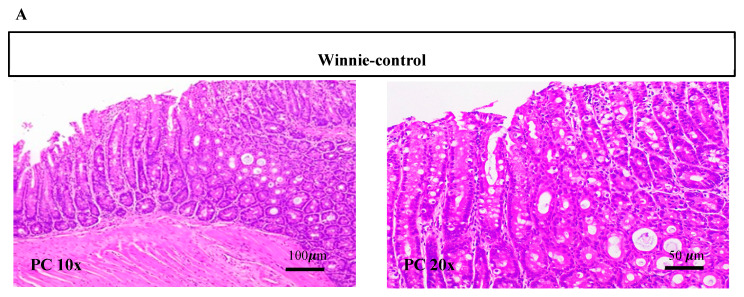

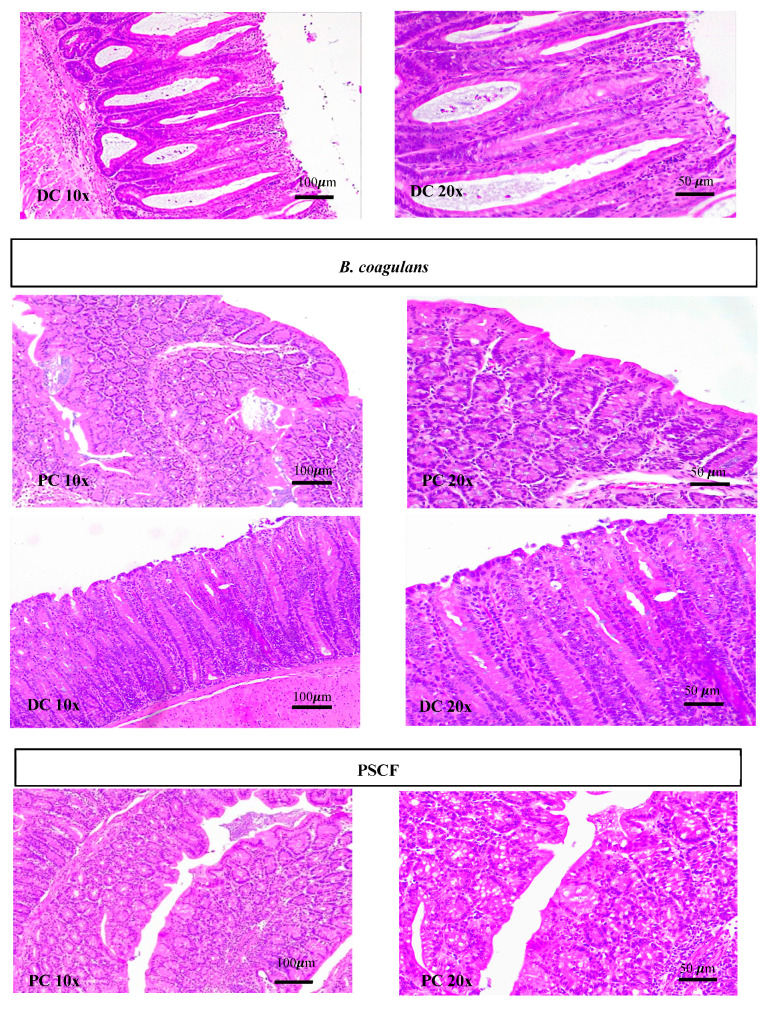

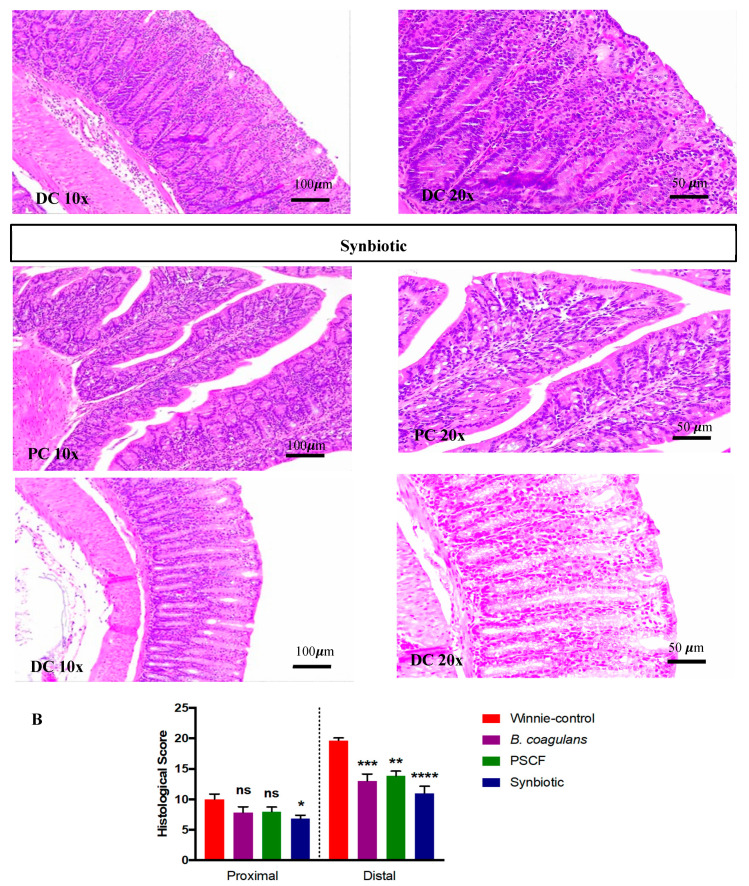
Effect of *B. coagulans* spores, PSCF and Synbiotic treatments on colon injury and inflammation in chronic-colitic-Winnie mice. (**A**) Histological images of proximal colon (PC) and distal colon (DC) tissues stained with hematoxylin and eosin at 10× and 20× for each experimental group. (**B**) Histological score calculated after microscopic analyses of proximal and distal sections of the colon. Results expressed as mean ± SEM (*n* = 6 per group), evaluated by one-way ANOVA (* *p* < 0.05, ** *p* < 0.01, *** *p* < 0.001, **** *p* < 0.0001).

Histological examination showed severe surface epithelial damage, goblet cell loss, crypt abscesses, crypt loss, distortion of crypt architecture, crypt hyperplasia and increased inflammatory infiltrate mostly affecting the distal colon (DC) section (Figure 3A) of Winnie-control compared with that of supplemented mice. Supplementation of Winnie with Synbiotic (11, *p* < 0.0001), *B. coagulans* (13, *p* = 0.0003) and PSCF (13.8, *p* = 0.0014) displayed significant improvements in the histology of the colon, particularly in the distal section compared with the marked histological alterations score of 19.7 in untreated Winnie-control mice (Figure 3B). The comparative histological score for proximal colon (PC) was also statically lower (*p* = 0.0443) in Winnie supplemented with synbiotic (6.83) compared to that of Winnie-control (10). *B. coagulans* (7.83, *p =* 0.2377) and PSCF (8, *p =* 0.3002) alone were not statistically effective in reducing the histological score in PC, thus supporting the necessity of the synergistic Synbiotic combination to achieve consistent benefits.

### 3.3. Synbiotic Supplementation Suppressed Colonic Pro-Inflammatory Cytokines

**Figure 4:**
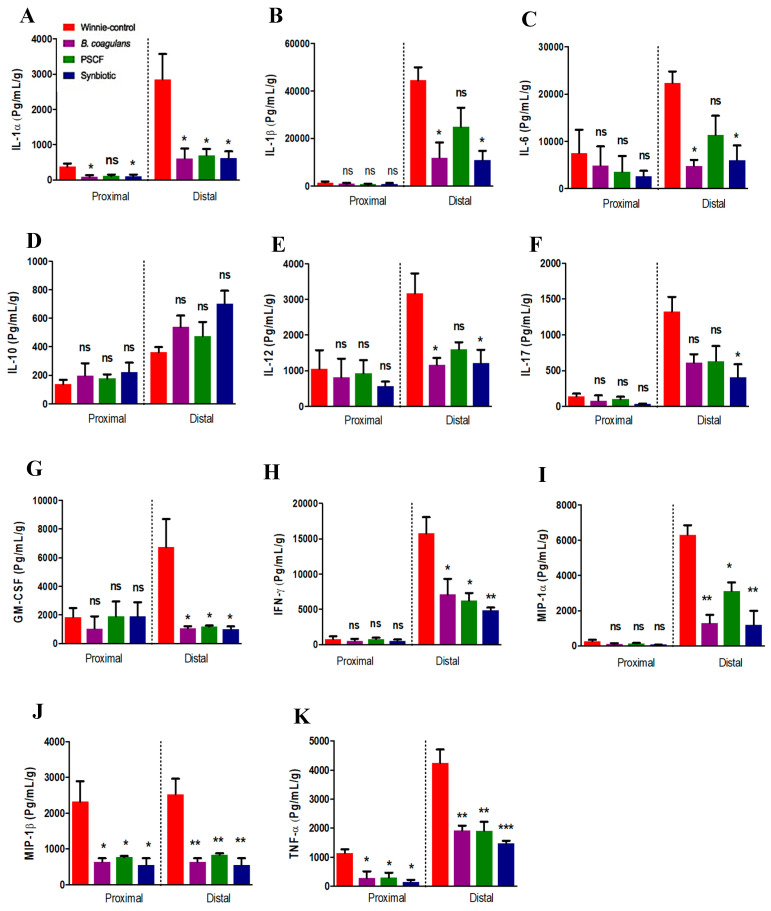
Effect of *B. coagulans* spores, PSCF and Synbiotic on immune markers in colon tissues. Protein levels of cytokines including (**A**) IL-1α, (**B**) IL-1β, (**C**) IL-6, (**D**) IL-10, (**E**) IL-12, (**F**) Il-17, (**G**) GM-CSF, (**H**) IFN-γ, (**I**) MIP-1α, (**J**) MIP-1β and (**K**) TNF-α in proximal and distal colon explants were analysed by Bio-plex. Statistical significance among groups evaluated by one-way ANOVA. * *p* < 0.05, ** *p* < 0.01, *** *p* < 0.001, ns-non-significant vs. Winnie-control and data expressed as mean ± SEM (*n* = 3 per group).

Cytokine analysis of the colonic tissue explants was used to ascertain the intestinal immunomodulatory and anti-inflammatory effects of *B. coagulans*, PSCF and their synbiotic combinations (Figure 4). The mucosal explants isolated from the colon of the untreated Winnie-control group showed marked secretion of a number of pro-inflammatory cytokines and chemokines in both PC and DC sections (Figure 4). Supplementation of Winnie with synbiotic markedly suppressed the level of the elevated pro-inflammatory mediators, particularly in the DC section compared with that of the *B. coagulans* and PSCF supplementations alone. In PC the *B. coagulans*, PSCF and Synbiotic groups showed significant reduction in levels of MIP-1β and TNF-α. While PSCF was effective in suppressing the levels of IL-1α (*p* = 0.0242), IFN-γ (*p* = 0.0179), GM-CSF (*p* = 0.0168), MIP-1α (*p* = 0.0204), MIP-1β (*p* = 0.0054), and TNF-α (*p* = 0.0022) in DC, no substantial reduction was noted for secretions of IL-1β (*p* = 0.1867), IL-6 (*p* = 0.1065), IL-12 (*p* = 0.0644) or IL-17 (*p* = 0.1044). Synbiotic and *B. coagulans* alone were statistically equivalent in suppressing the secretions of IL-1α (*p* = 0.0203, 0.0198, respectively), IL-1β (*p* = 0.0195, 0.0229, respectively), IL-6 (*p* = 0.0173, 0.0116, respectively), IL-12 (*p* = 0.0225, 0.0198, respectively), GM-CSF (*p* = 0.0139, 0.0148, respectively), MIP-1α (*p* = 0.0012, 0.0014, respectively), and MIP-1β (*p* = 0.0020, 0.0027, respectively) in DC. However, Synbiotic compared to *B. coagulans* supplementation was more potent in reducing the levels of IL-17 (*p* = 0.0304, 0.1044, respectively), IFN-γ (*p* = 0.0084, 0.0292, respectively), and TNF-α (*p* = 0.0007, 0.0023, respectively). Moreover, Synbiotic supplementation elevated the anti-inflammatory IL-10 level in DC, although this was not statistically significant (*p* = 0.0668).

### 3.4. Synbiotic Supplementation Promoted Systemic Anti-Inflammatory Effects

**Figure 5:**
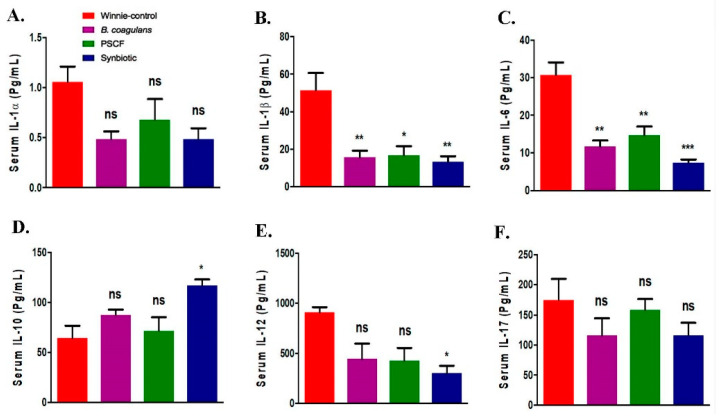

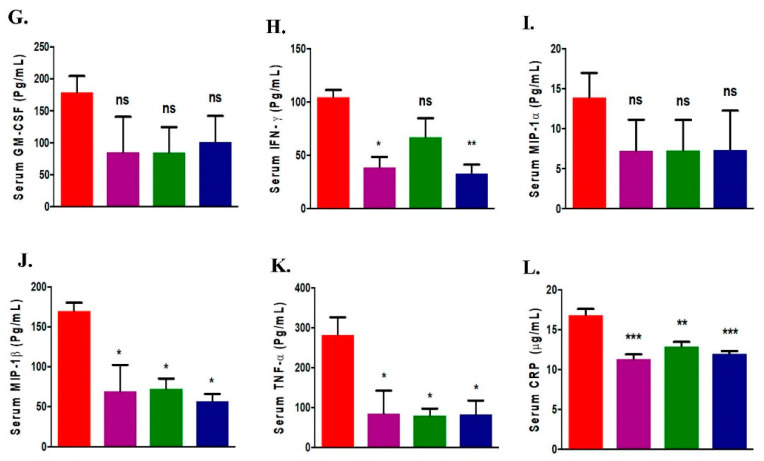
Effect of *B. coagulans* spores, PSCF and synbiotic on immune markers in serum. Protein levels of cytokines including (**A**) IL-1α, (**B**) IL-1β, (**C**) IL-6, (**D**) IL-10, (**E**) IL-12, (**F**) IL-17, (**G**) GM-CSF, (**H**) IFN-γ, (**I**) MIP-1α and (**J**) MIP-1β and (**K**) TNF-α in serum were analysed by Bio-plex. CRP levels in serum (**L**) measured by ELISA. Statistical significance among groups evaluated by one-way ANOVA. * *p* < 0.05, ** *p* < 0.01, *** *p* < 0.001, ns = non-significant vs. Winnie-control and data expressed as mean ± SEM (*n* = 3 per group).

Substantial immune regulatory effects of the supplementations in Winnie were noted for most serum cytokines tested except for IL-1α, IL-17, GM-CSF and MIP-1α (Figure 5A–K). *B. coagulans* and PSCF supplementations alone suppressed the elevated serum levels of IL-1β (*p* = 0.0094, 0.0110, respectively), IL-6 (*p* = 0.0015, 0.0045, respectively), IFN-γ (*p* = 0.0156, 0.1739, respectively), MIP-1β (*p* = 0.246, 0.0288, respectively) and TNF-α (*p* = 0.0384, 0.0338, respectively) in chronic-colitic-Winnie. In addition to suppressing these cytokines, Synbiotic supplementation showed relatively more profound suppression in the levels of IL-6 (*p* = 0.0004) and IFN-γ (*p* = 0.0099) further supporting the existence of synergetic beneficial effects. Moreover, compared to Winnie-control, synbiotic significantly elevated the anti-inflammatory IL-10 levels in serum (*p* = 0.0233). It was more effective than *B. coagulans* (*p* = 0.4021) and PSCF (*p* = 0.9481) alone. Marked systemic immunomodulatory outcome effects of the supplementations in chronic colitic Winnie was also evidenced by the ability of *B. coagulans*, PSCF and its synbiotic combination to reduce the elevated CRP in the serum (11.32 ± 0.58, 12.91 ± 0.57 and 12 ± 0.32 μg/mL, respectively) compared to that in unsupplemented Winnie-controls (16.81 ± 0.80 μg/mL) as depicted in Figure 5L. These observations support the substantial immunomodulatory and anti-inflammatory efficacies of the supplementations used in the study to reduce colonic and systemic inflammation in chronic colitis.

### 3.5. Synbiotic Supplementation Improved Microbial Diversity Associated with Chronic Gut Inflammation

**Figure 6:**
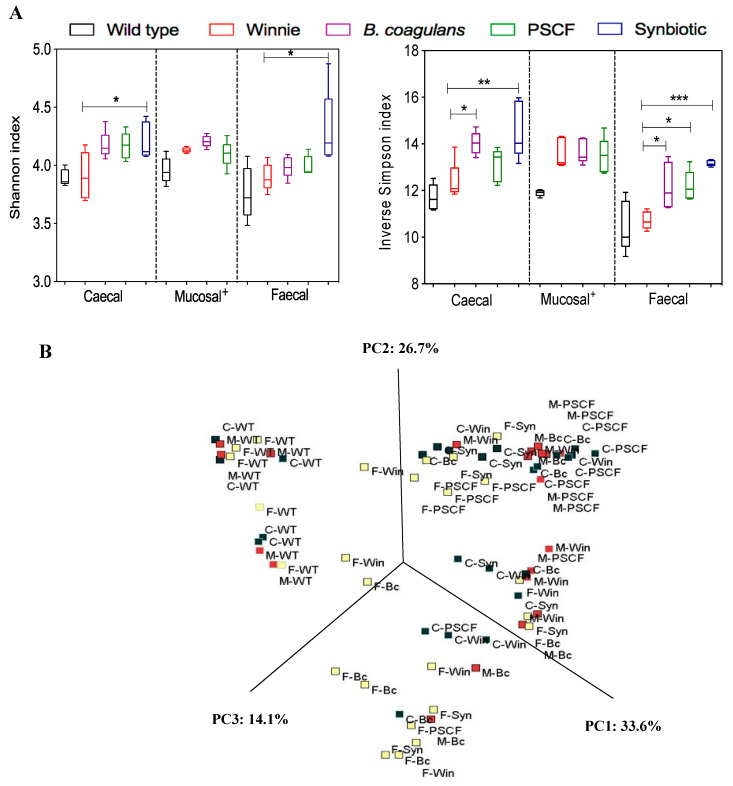
Effect of supplementations on microbial diversity amongst caecal (C-), mucosal-associated (M-) and Faecal (F-) contents of wild-type (WT), Winnie-control (Win), *B. coagulans* (Bc) spores, PSCF and Synbiotic (Syn) groups. (**A**) Comparison of Alpha (α) diversity indices. Statistical significance among groups evaluated by one-way ANOVA. * *p* < 0.05, ** *p* < 0.01, *** *p* < 0.001 Versus Winnie-control group (+ = mucosal Synbiotic sample data could not be determined). Data expressed as mean ± SEM (*n* = 5 per group). (**B**) Principal component analysis (PCoA) plot based on Bray-Curtis distances.

Taxonomic and functional profiles of 75 samples (*n* = 5 per group), including the caecal, mucosal-associated and faecal samples of WT and Winnie groups, were generated using the 16 S rRNA gene sequencing-based method. In the experiment, 3 out of 5 synbiotic mucosal-associated samples collected did not generate the required 30,000 minimum sequencing raw read outputs. Hence, the effect of synbiotic in modulating microbial diversity in mucosal-associated samples could not be determined. The effects of supplementation of diet with *B. coagulans*, PSCF and synbiotic in modulating microbial alpha and beta diversities in chronic-colitic-Winnie across caecal, mucosal-associated and faecal contents were assessed (Figure 6). The supplementations caused substantial increases in alpha diversity indices and the effect varied across the sample types (Figure 6A). PCoA plots of phylogeny with Bray-Curtis ecological indexing using ward clustering (Figure 6B) showed distinct demarcation of WT group from that of Winnie groups (both supplemented and unsupplemented) with three distinct clusters at the operational taxonomic units (OTU) level. This indicated clear differences in the microbial patterns between the healthy WT and the inflamed Winnie colitic mice. However, the microbial communities (irrespective of the sample types) of Winnie-control and supplemented Winnie groups were scattered, with no clear distinction between groups. This suggests high inter-individual variability among the supplemented and unsupplemented Winnie mice in terms of microbial diversity.

### 3.6. Synbiotic Supplementation Reduced Dysbiosis Associated with Chronic Inflammation in Winnie

**Figure 7:**
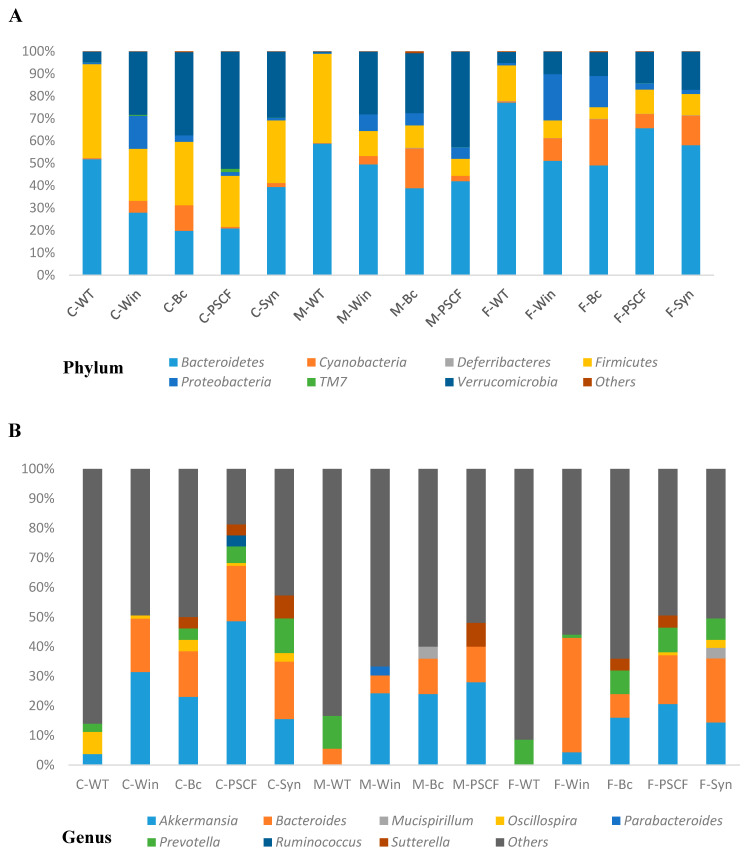
Relative abundances (%) of caecal (C)-, mucosal (M)- and Faecal (F)- associated microbiota at (**A**) phylum and (**B**) genus level observed in wild-type (WT), Winnie-control (Win), *B. coagulans* (Bc) spores, PSCF and Synbiotic (Syn) groups; (*n* = 5 per group).

Figure 7A indicates the phylum-level changes in the caecal, mucosal-associated and faecal samples of WT and Winnie mice, which are dominated by *Bacteroidetes* and *Firmicutes* and moderately by *Verrucomicrobia.* Around 99% of the total microbial abundance was classified into seven major phyla (*Bacteroidetes*, *Cyanobacteria*, *Deferribacteres*, *Firmicutes*, *Proteobacteria*, *TM7* and *Verrucomicrobia*) in all sample types, while the rest were allocated as unclassified or others. Although Winnie mice shared most of the same phyla as healthy WT, levels of their abundance varied. While WT caecal and faecal samples showed 43% and 16%, respectively, of relative abundance of *Firmicutes*, their levels were reduced in Winnie-control to only 16% in caecal and 8% in faecal samples. Similarly, the phylum *Bacteroidetes* was also reduced in Winnie-control (19%) caecal samples compared to that of WT (54%). Though *B. coagulans* and PSCF supplementations resulted in elevation of *Firmicute* levels (28% and 22%, respectively), no effect was observed for relative abundance of *Bacteroidetes* (19% and 20%, respectively). Synbiotic supplementation, however, increased *Firmicutes* (25%) and *Bacteroidetes* (36%) levels relative to that of Winnie-control in the caecum. Moreover, in faecal samples, Synbiotic supplementation was effective in inducing modulations in the levels of *Firmicutes* (25%) and *Bacteroidetes* (36%) compared with that in the Winnie-control (7.9% and 51%, respectively). In contrast to WT, all samples in Winnie groups showed increased abundance of *Verrucomicrobia*. As shown by LEfSe analysis (Supplementary Figure S1), among the Winnie experimental groups, PSCF supplementations caused substantial increase in *Verrucomicrobia* levels in caecal (52%) followed by in mucosal-associated (42%) and faecal (13%) samples. Similarly, compared to the spike in the level of *Proteobacteria* phylum in Winnie-control faecal samples (20.5%), Synbiotic suppressed the level (1.9%) similar to that in WT (1.1%). Among the other minor phyla, *TM7* (0.32% in Winnie-control) in caecum were suppressed by synbiotic (0.057%) and *B. coagulans* (0.022%) and were closer to the levels observed in WT (0.036%). PSCF, however, increased TM7 levels in caecal samples (1.39%).

At the genus level, the distribution of site-specific microbial populations of Winnie-control was markedly different when compared to WT (Figure 7B). While WT caecal samples showed the presence of *Oscillospira*, it was remarkably reduced in cecum of Winnie-Control. *B. coagulans* supplementation increased the abundance of *Oscillospira* in caecum, *Akkermansia* in faeces while modulating *Bacteroides* in faecal samples compared to that of Winnie-control. PSCF supplementation markedly enriched *Akkermansia* in caecal, faecal and mucosal-associated samples compared with that of in Winnie-control. Synbiotic supplementation in Winnie not only favoured the abundance of *Bacteroides* in faeces as revealed by LEfSe analysis (Supplementary Figure S2), it was also observed to increase *Oscillospira* in caecal and faecal samples. Winnie-control showed complete absence of *Oscillospira* in mucosal and faecal samples. While *Prevotella* showed its presence in caecal, mucosal-associated and faecal samples of WT, their levels were undetected in caecal and mucosal-associated samples of Winnie-control, while very low levels (1%) were detected in faecal samples. *B. coagulans*, PSCF and Synbiotic supplementations, however, were able to induce appreciable increase in *Prevotella* levels in Winnie colitic mice. At the species level (Supplementary Figure S2), while WT samples showed the presence of *Ruminococcus gnavus* in all sample types, it was at very low levels in unsupplemented Winnie-control and supplemented Winnie groups. Compared to WT samples, Winnie samples showed increased prevalence of *Akkermansia muciniphila*. The ability of PSCF to substantially elevate the abundance of *A. muciniphila* in caecal samples was confirmed by LEfSe analysis (Supplementary Figure S1). PSCF also modulated their levels in mucosal-associated and faecal samples, while Synbiotic was effective in increasing their level in faecal samples the most. High levels of *Desulfovibrio C21_c20* in faecal samples of WT and Winnie-control were greatly reduced with *B. coagulans*, PSCF and Synbiotic supplementations. Compared to WT, Winnie-control samples showed increased *Bacteroides uniformis*, while its level was suppressed by Synbiotic. *Bacteroides distasonis* remained undetected in the WT samples, while its presence was detected in Winnie-control samples. The levels of these species were reduced marginally by Synbiotic supplementation in caecal samples while *B. coagulans* suppressing their level in faecal samples minorly. Additionally, *Eubacterium dolichum,* that were at undetectable levels in samples from WT mice, had a notable prevalence in the samples of Winnie-control but their levels were reduced by Synbiotic and PSCF supplementations. *B. coagulans* supplementation alone did not affect the abundance of these bacteria.

### 3.7. Synbiotic Supplementation Induced Augmented Production of SCFAs along the Colon

**Figure 8:**
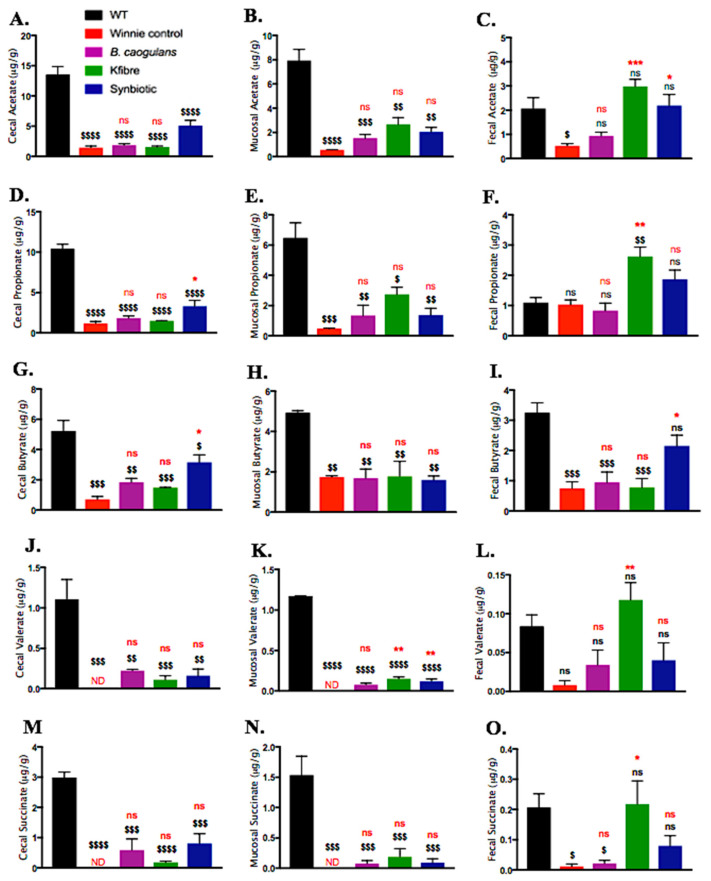
Effects of *B. coagulans* spores, PSCF and Synbiotic in modulating SCFA concentrations in caecal, mucosal-associated and faecal contents in Winnie vs. Wild-type (WT) mice. Caecal-acetate (**A**), propionate (**D**), butyrate (**G**), valerate (**J**), succinate (**M**); mucosal-associated acetate (**B**), propionate (**E**), butyrate (**H**), valerate (**K**), succinate (**N**) and faecal-acetate (**C**), propionate (**F**), butyrate (**I**), valerate (**L**), succinate (**O**). Statistical significance among groups evaluated by one-way ANOVA. * *p* < 0.05, ** *p* < 0.01, *** *p* < 0.001, ns vs. Winnie-control group and ^$^ *p* < 0.05, ^$$^ *p* < 0.01, ^$$$^ *p* < 0.001, ^$$$$^ *p* < 0.0001, ns vs. WT group. Data expressed as mean ± SEM (*n* = 5 per group). ns = non-significant, ND = not detected.

Feeding Winnie mice with PSCF, Synbiotic and to a lesser extent *B. coagulans* supplementations induced substantial modulations in the SCFA concentrations and their effects varied across caecal, mucosal-associated and faecal contents (Figure 8). Overall, higher concentrations of SCFAs were noted in caecal contents than in mucosal-associated and faecal contents. Compared to the SCFAs concentration in WT mice, unsupplemented Winnie-control mice exhibited marked depletion in acetate, propionate and butyrate, while valerate and succinate were at undetectable levels. Although the supplementations were not statistically effective in normalising the whole SCFAs profile in range with that of WT, the supplementations substantially induced elevation in the tested SCFAs concentration relative to that of Winnie-control. While *B. coagulans* was ineffective (*p* > 0.005) in elevating the plummeted SCFAs levels, PCSF alone showed ability to significantly (*p* < 0.005) increase acetate, propionate, valerate and succinate in faecal contents. In caecal contents, Synbiotic supplementation compared to Winnie-control resulted in a significant increase in concentrations of acetate (5.07 ± 0.9, 1.45 ± 0.3 μg/g, respectively), propionate (3.29 ± 0.7, 1.15 ± 0.3 μg/g, respectively) and butyrate (3.16 ± 0.5, 0.707 ± 0.2 μg/g, respectively). Moreover, in faecal contents, synbiotic exhibited the excellent ability to not only increase the levels of acetate (2.19 ± 0.4 μg/g), propionate (1.87 ± 0.3 μg/g) and butyrate (2.15 ± 0.4 μg/g) in Winnie, the levels were equivalent to that of WT mice (2.06 ± 0.4, 1.09 ± 0.2, 3.25 ± 0.3 μg/g, respectively). Furthermore, only Synbiotic supplementation was effective in elevating butyrate levels along the entire length of colon (in caecal and faecal samples) relative to PSCF and *B. coagulans* alone. This finding indicates the prudence of application of synergetic Synbiotic components to provide elevated butyrate levels along the whole length of colon.

## 4. Discussion

Abnormally reduced SCFA-producing bacteria and SCFA levels in the gut are amongst the key indicators of dysbiotic patterns in IBD. The SCFAs, produced via fermentation of undigestible DF, are one of the primary mediators by which gut microbiome affects host physiology and disease modulation. This is reflected by the ability of SCFAs in influencing gut barrier function and immune regulation [2,40]. This study provides a proof-of-concept data on benefits of compatible synergistic synbiotic supplementation to induce modulation of gut environment in IBD via multifaceted approach. By strategically applying a whole-plant complex DF alongside probiotic strain known to metabolise the plant substrate to generate fermentation products, we demonstrated the therapeutic efficacy of such synergy in resolving IBD inflammation. The synergistic potency of synbiotic in improving clinical manifestations, reducing colonic histological damage, induce immune regulation and modulations in gut microbiota could be largely attributed to the augmented SCFA production.

A dietary strategy involving the supplementations of *B. coagulans*, PSCF and their synbiotic combination was investigated in ameliorating chronic colitis in the spontaneous chronic-colitic-Winnie model. In Winnie mice, spontaneous chronic colitis results from a primary intestinal epithelial defect conferred by a missense mutation in the *Muc2* mucin gene [28]. This defect leads to alterations in epithelial barrier function resulting in aberrant pro-inflammatory immune response. This accompanies diarrhoea, ulcerations, rectal bleeding and weight loss in Winnie similar to clinical IBD [28,29,41]. The excellent ability of *B. coagulans*, PSCF and Synbiotic supplementations in the current study, in attenuating gradual rise in DAI, body weight loss and excretion of diarrheic/bleeding faeces (Figure 2) indicate benefits of the tested dietary strategies. This is in agreement with our previous study confirming amelioration of chemically-induced acute colitic clinical manifestations by these dietary supplementations [27]. The beneficial effect imparted by synbiotic supplementation could be related to the synergistic actions between the bioactive components and supports its potential application in reducing diarrheal episodes in clinical IBD.

The alterations in the colonic epithelium is a hallmark of IBD [3]. These alterations lead to disturbances in gut barrier function resulting in translocation of the intestinal microbiota and activation of immune system leading to aggravation of the disease. The augmented beneficial effects of the synbiotic application, compared to that of *B. coagulans* and PSCF alone, was evidenced by its ability to decrease the histological scores in both proximal and distal colon sections of the mice (Figure 3B). The colonic histological damage mostly to the distal region in Winnie has been previously shown [28,30,41]. The ability of synbiotic treatment in reducing the colonic surface epithelial damage (Figure 3A) could be correlated with the improvement in the clinical manifestations in chronic-colitic-Winnie. Such synergetic beneficial outcomes of the Synbiotic combination could be accounted for by the reinforcing of weakened colonic barrier integrity in chronic colitis.

Disruption in the colonic barrier integrity in IBD exacerbates dysregulated immune responses leading in an inflammation cascade and tissue damage [42]. In the current study, unsupplemented Winnie-control colon were determined to secrete elevated levels of pro-inflammatory cytokines and chemokines (Figure 4) in agreement with previous report [30]. *B. coagulans* and PSCF treatment alone were able to reduce most of these elevated cytokines. However, Synbiotic supplementation exhibited more consistent effects in suppressing the secretion levels of these pro-inflammatory cytokines and chemokines in chronic-colitic-Winnie colon, thus, supporting its potentiated synergistic immune-modulatory efficacy in the inflamed tissue.

Increase in systemic pro-inflammatory cytokines is also associated with clinical IBD [43]. The capability of Synbiotic for imparting beneficial systemic anti-inflammatory effects was evidenced by its ability to suppress the levels of pro-inflammatory cytokines and chemokines in serum while also increasing anti-inflammatory IL-10. IL-10 downregulates antigen presentation and subsequent release of proinflammatory cytokines, leading to attenuation of mucosal inflammation [42]. Furthermore, all supplementations were effective in reducing the elevated serum CRP levels in Winnie. High levels of CRP are reported in human IBD patients [43]. CRP production in the liver and its release in the blood stream is stimulated by circulating IL-6 during inflammation. The marked ability of the supplementations in our study to reduce serum IL-6 and CRP levels supports their potential application in IBD to induce anti-inflammatory and immunomodulatory effects to hinder the inflammatory cascade. The amplified ability of Synbiotic for improving the overall pro-inflammatory profile of Winnie, could be attributed to either a direct immune-regulating effect of *B. coagulans* and PSCF, and/or due to their effect on improvement of colonic epithelial integrity. Either of these effects could lead to a decrease in luminal antigen load and optimal activation of innate immune system. The potentiated efficacy of synbiotic in immune-modulation and gut epithelium improvement in our study, could also be correlated to augmented SCFA levels and microbiota modulations.

Interaction of gut microbiota with host is largely mediated via its metabolic products. Microbial SCFAs are the key mediators that affect the IBD pathology. They exert benefits through enhancing gut epithelium and immunoregulation [2]. Recently, the DF-mediated tumour-suppressive effect was shown to be conferred in a microbiota- and butyrate-dependent manner in mice harbouring butyrate-producing bacterium [14]. In contrast, fibre failed to reproduce benefits in mice colonised with mutant strain of butyrate-producing bacterium. The findings highlight the importance of maintaining and/or replenishing the fibre-digesting microbiota in improving gut health. Supporting the growth of secondary fermenters in the gut by DF administration for augmentation of SCFAs has also been demonstrated [44]. In corroboration, application of synergistic synbiotic application in our study targeted at the modulation of gut microbiota and concurrent augmented SCFA levels along the colon demonstrated benefits. In addition, synbiotic efficacy in influencing the immune response and colonic epithelial integrity indicate the prudence of synergism among the synbiotic components tested.

Human clinical studies on the changes in microbiota associated with IBD are normally restricted to faecal sampling only. The dysbiosis associated with IBD, however, may not be limited only to the faecal microbiota as the microbial numbers and composition vary along the gastrointestinal tract. Mounting evidence has indicated a distinction between the microbial dysbiotic pattern in different locations along the gastrointestinal tract of IBD patients [45,46]. The current study confirmed site-specific microbial and SCFA shifts along the caecal, mucosal-associated and faecal samples. In addition, differences in microbiota and SCFA levels were also recorded between unsupplemented Winnie-control, supplemented Winnie and WT mice. Notable differences in the caecal, mucosal-associated and faecal microbiota of WT and Winnie mice were evident at levels of bacterial taxonomical classification, including the phylum, genus and species. These observations agree with previous reports that confirmed distinct microbial patterns in faecal samples of inflamed Winnie and healthy WT mice [29]. Although Winnie mice shared most of the same phylum as healthy WT mice, levels of their abundance were markedly different.

Dysbiosis of gut microbial communities has been well recognized as one of the hallmarks of pathogenesis in IBD. Depletion of commensally associated bacteria, notably members of the phyla *Bacteroidetes* and *Firmicutes*, has been linked with IBD in several clinical reports [40,46,47] and Winnie mice [29]. Compared with Winnie-control, Synbiotic supplementation elevated the levels of *Bacteroidetes* and *Firmicutes* in chronic colitic-Winnie. The genus *Prevotella*, belonging to phylum *Bacteroidetes*, was also significantly declined in the Winnie-control with a low presence only in faecal samples, but its prevalence was enhanced by synbiotic supplementation in both the caecum and faeces. A high fibre diet has been linked to increased prevalence of *Prevotella* in healthy human subjects [48] and in African children consuming high-fibre, low-fat diets [49]. *Prevotella* is a well-known dietary fibre fermenter for production of SCFAs [50]. *Prevotella* species are also known to possess enzymes for degradation of an array of polysaccharides including cellulose, hemicellulose and xylans [49,51]. From this view, the increase in *Prevotella* levels in PSCF-supplemented Winnie could be correlated to the high content of plant cell wall fractions available for degradation. The ability of *B. coagulans* supplementation to also influence the abundance of *Prevotella* indicates a potential beneficial effect of the probiotic. The combined beneficial effect of increasing the prevalence of *Prevotella* and elevation of SCFA levels in Winnie by synbiotic supplementation suggests synergistic functioning.

The decreased prevalence of butyrate-producing *Firmicutes* is often associated with IBD [2,52]. Members of genus *Oscillospira* of *Firmicutes* are butyrate-producers [48]. They were detected in cecum of WT but there was a complete absence in Winnie-control (except at small percentage in faeces). The *Oscillospira* level was found to be severely decreased in IBD patients [53,54]. Synbiotic supplementation effectively recovered the altered levels of *Oscillospira* in caecum and faeces, while *B. coagulans* recovered *Oscillospira* in cecum. *Oscillospira* are reported to be unlikely to degrade complex fibres, instead relying on fermentation products secreted by other species, particularly *Bacteroides* or on host mucin glycans [52]. In this context, recovery of *Oscillospira* population by synbiotic suggests its influence via cross-feeding. Synbiotic supplementation also influenced the level of *Blautia* of *Fimicutes* Phylum (Supplementary Figure S2) that has been reported in healthy Chilean individuals [48] and is one of the butyrate-producing bacteria in the human gut [47].

Increased prevalence of *Proteobacteria* is considered a potential diagnostic signature of dysbiosis and risk of inflammation. Commensal *Proteobacteria* members are known to increase their endurance during dysbiosis-driven selective pressure [55]. Relative to Winnie-control, Synbiotic was also more effective in reducing the abundance of phylum *Proteobacteria* in caecal and faecal samples compared with either *B. coagulans* or PSCF supplementation. *B. coagulans* and PSCF had no effect on *Proteobacteria* level in mucosal-associated samples. A significant increase in members of *Proteobacteria* phylum has been previously reported in faeces and in the caecal lymphoid patches of Winnie [29]. Abundance of *Desulfovibrio C21_c20* species belonging to *Proteobacteria* phylum were also reduced in Winnie supplemented with Synbiotic. The rates of hydrogen sulfide production were higher among the sulfate-reducing bacteria isolated from patients with UC compared to those in healthy volunteers [56]. Species of genus *Desulfovibrio* are known to inhibit epithelial butyrate metabolism via release of hydrogen sulfide [57]. These observations could be related to the decline in butyrate-producing bacteria and reduced butyrate production in inflamed colitic Winnie-control mice (Figure 8). The adversity of such inhibitory effect could also be reflected by the damage to the colonic epithelium in Winnie. The outgrowth of *Proteobacteria* in European children consuming a low-fibre, high-fat diet, compared to African children consuming high-fibre, low-fat diet, was reported by De Filippo et al. [49]. In this context, the influence of Synbiotic in suppressing the increased prevalence of *Proteobacteria* in Winnie in the current study, could be attributed to the high whole-plant fibre content of PSCF. Resolution of dysbiosis by enrichment of the fibre-digesting gut bacterial members is posit to exert selective pressure on members of *Proteobacteria* [55].

High abundance of *Verrucomicrobia* members (*Akkermansia*) has been reported in healthy Chilean subjects [48] while its decreased abundance is noted in IBD patients [58,59,60]. Interestingly, compared to that of WT, increased abundance of *Verrucomicrobia* was evident in all Winnie groups irrespective of supplementation/non-supplementation and the sample types. Members of genus *Akkermansia* and species *A. muciniphila* were also elevated by PSCF supplementation in this research, specifically in the caecum. Additionally, in faecal samples all the three supplementations caused a moderate increase in *Akkermansia*. The efficient colonisation of *A. muciniphila* in the caecum is attributed to the high mucin production [61]. The increased ability of PSCF to induce growth of *Akkermansia* could be related to its indigestible fibre content [62]. Dietary polyphenols have been determined to promote growth of *Akkermansia* and were strongly correlated with the improvement of inflammation in DSS-induced colitis [63] and high-fat diet fed mice [64,65]. *A. muciniphila* is a propionic-inducing bacterium that uses mucin as nutrients [61]. The increased prevalence of *Akkermansia* in Winnie mice, relative to that in WT mice, is intriguing considering the less mucin being secreted, owing to the point mutation in *Muc2* gene of Winnie [28]. The bloom in the members of genus *Akkermansia* in Winnie could be partially related to its ability to metabolize the fatty acid hexadecenoic acid, which has been reported earlier to be heightened 2–3 fold compared with that in WT mice [29]. Moreover, the aerotolerant ability of some species of *Akkermansia* confers resistance to the oxidative environment in the inflammatory colon [65,66]. In substantiation of our observation, increased abundance of *Akkermansia* was also reported in DSS-induced colitis [67]. Therefore, there seems to be no clear consensus on the role of *Akkermansia* in chronic gut inflammation in IBD animal models. In contrast however, *A. muciniphila* is known as a modulator for gut homeostasis [61] and is abundantly present in healthy human intestinal tract making up 1–4% of the bacterial population in the colon [68,69]. A recent study has demonstrated improvement in metabolic parameters in over-weight and obese human subjects by supplementation with *A. muciniphila* [70]. Decline in its abundance is reported in human IBD patients suggesting its potential anti-inflammatory role [58,59]. A beneficial effect of *Akkermansia* on colitis, however, is effected by its extracellular vesicles that were found to protect against DSS-induced colitis [71]. Moreover, besides being able to degrade mucins, *Akkermansia* was also found to increase mucus layer thickness in prebiotic treated diet-induced obese mice, suggesting its potential ability to stimulate mucin synthesis [72]. *Akkermansia* has additionally been demonstrated in-vitro to adhere to and restore the integrity of the epithelial cell layer, while no adherence was observed to human mucus thus, suggesting that the beneficial role of this bacterium in the gut is not exclusively associated with mucus layer physiology [66]. Considering that the attenuation of colitic inflammatory parameters induced by PSCF and synbiotic supplementations was associated with a significant increase in *Akkermansia* in Winnie, a beneficial effect on gut inflammation is indicated.

Reduced SCFA levels are an important indicator of dysbiosis in IBD. Dysregulation in microbiota-derived SCFA production is often implicated with dysbiosis in IBD and therefore, has gathered considerable research interest [2]. The Synbiotic supplement showed marked efficacy for modulating the altered SCFA production in colitic-Winnie mice (Figure 8). SCFAs that are solely metabolized by gut bacteria from indigestible carbohydrates from fibre-rich diets, have been affirmed to attenuate disease severity in animal models and clinical UC [2]. Consistent with the previous Winnie report [29], significant decline in the SCFA levels were detected in unsupplemented Winnie-control mice compared to that in healthy WT mice. The altered SCFA production in Winnie could be associated with the decline in the abundance of SCFA producing bacteria belonging to genera *Oscillospira* and *Prevotella* as observed in Winnie-control group in the current study (Figure 7). The consistently high ability of synbiotic supplementation to address the pathology caused by the Winnie mutation could be evidenced by its ability to elicit SCFA production in caecal and faecal samples thus, mediating a trophic effect along the entire colon length. This observation could potentially be correlated with the increased butyrate-producing *Oscillospira* genus with Synbiotic but was not detected in Winnie-control mice. Additionally, the ability of Synbiotic supplementation to promote the abundance of SCFA-producing *Prevotella* genus could be associated with the elevation in the SCFA levels. Butyrate serves as the primary energy source for colonocytes and mediates regulation of cytokines further, imparting protection against inflammation in IBD [2]. Although, *B. coagulans* supplementation alone could not confer any substantial mediation in the SCFA production compared to Winnie-control, PSCF supplementation triggered elevations in the level of acetate and propionate in the faeces. However, this effect was not observed for the butyrate level. The propionate boosting effect of PSCF alone, could be correlated to its ability in inducing bloom in relative abundance of *Akkermansia* in Winnie (Figure 7). In in-vitro organoid testing, *A. muciniphila* was shown to induce substantial concentrations of propionate and acetate but not butyrate [73], in alignment with the observations of the current study. Its mucin degrading activity is known to mediate the production of propionate and acetate [74]. Butyrate has been confirmed to increase epithelial integrity [2] consistent with the improvement in histological and immune-regulatory observations induced by Synbiotic in the present study. In addition to butyrate, acetate and propionate bind to certain metabolite-sensing G-protein-coupled receptors (such as GPR43, GPR109A), subsequently inducing a beneficial immune response and improving epithelial integrity [2]. Valerate, that was studied to promote intestinal growth and reduce inflammatory pathogenesis in colitis and cancer [75], was increased by PSCF supplementation. The high SCFA levels induced by Synbiotic supplementation in Winnie owing to the synergistic combination could be accounted for excellent immuno-modulatory effects confirmed in the present study.

The employment of a suitable probiotic bacteria targeted at metabolising the compatible prebiotic fibres to elevate SCFA production is a pragmatic synbiotic strategy towards resolving IBD inflammation. In the current study, relative to the individual supplementations, the synergistic synbiotic supplementation, not only induced increased levels of acetate, propionate and butyrate along the entire colon length, but the SCFA levels in the faecal samples were considerably restored to a level similar to that in WT-mice. The inability of *B. coagulans* to modulate the SCFA production in this chronic-colitic-Winnie model, indicates two possible inferences. Firstly, a possible lack of available fermentable fibre in normal chow diet to be directly metabolized by this probiotic and secondly, its compromised efficiency in promoting microbial growth of SCFA producers in inflamed Winnie colon. The excellent SCFA induction efficacy of *B. coagulans* in synbiotic combination with PSCF, suggests its ability to metabolise the fibre fractions to induce beneficial modulatory outcomes. The *B. coagulans* possess excellent capacity to ferment a variety of plant fibres [22,23]. Thus, the efficacy of *B. coagulans* in fermenting complex plant cellular polysaccharides, such that present in PSCF, makes them an ideal bioactive combination for synbiotic application in conferring trophic effects of SCFAs in IBD along the entire colon length. SCFAs are known to induce immune-modulation by engaging with GPRs, leading to direct local and to systemic anti-inflammatory effects [2] as evidenced by the improved cytokine profile in the current study. These observations merit the application of synergistic synbiotic combination to achieve potentiated benefits in resolving the recurrent inflammatory pathophysiological circuit in IBD.

## 5. Conclusions

The findings of this study highlight a significant efficacy of synergistic synbiotic supplementation in ameliorating the chronic colitis as evidenced by attenuation of spontaneous colitis in mice model of IBD. The results have demonstrated potentiated anti-inflammatory outcome effects of synbiotic treatment supplementation carrying whole-plant PSCF and *B. coagulans* spores by reducing clinical manifestations, colonic damage and inflammatory mediators, while modulating the gut microbiota and SCFA profiles of chronic colitic mice. The observations support the hypothesis that supplementation of PSCF and *B. coagulans,* known to metabolise the indigestible fibres, together produced a synergistic combination that augmented the beneficial outcome effects against chronic colonic inflammation. The study underscores the value of systematically delineating the mechanistic functioning of synbiotic ingredients to screen and develop potent novel synbiotic treatments for targeted human health application. The study also emphasised the application of synergistic synbiotic as a two-point approach in suppressing the overall inflammation profile by targeting different mechanistic approaches to resolve the recurrent inflammatory cycle as evidenced in this murine IBD model. The significant therapeutic effects of *B. coagulans* and PSCF in a synbiotic combination, evidenced in this study, warrants testing in human IBD trials to mitigate inflammation as an adjuvant therapy.

## 6. Patents

The information from this study has been filed as a provisional patent application in Australia titled “Preparation for the Treatment of Inflammatory Bowel Disease using a Whole Plant Fibre Extract from Sugar cane” with application number 2018902145 (filing date of 15 June 2018) and now at PCT stage (PCT/AU2019/050604). Information relating to novelty of synergy between probiotic *Bacillus coagulans* and prebiotic whole plant sugar cane fibre in imparting health benefits is the subject of the patented claim.

## Supplementary Materials

The following are available online at https://www.mdpi.com/2072-6643/12/8/2380/s1, Figure S1: Biomarker analysis with Linear Discriminant Analysis (LDA) Effect Size (LEfSe) scoring plot using Kruskal-Wallis rank sum test. Figure S2: Relative abundances (%) of caecal (C)-, mucosal (M)- and Faecal (F)-associated microbiota at species level. Table S1: Nutritional information of KfibreTM Prebiotic Sugarcane Fibre, Table S2: Nutritional information of mice standard chow diet.
