# Supplementary material for: Modulating the Microbiome and Immune Responses Using Whole Plant Fibre in Synbiotic Combination with Fibre-Digesting Probiotic Attenuates Chronic Colonic Inflammation in Spontaneous Colitic Mice Model of IBD
Source: Nutrients. 2020 Aug 9;12(8):2380. doi: 10.3390/nu12082380 (PMC7468978; doi:10.3390/nu12082380)
Supplement: Supplementary file 1 [file nutrients-12-02380-s001.pdf]

Modulating the microbiome and immune responses using whole plant fibre in synbiotic combination with fibre-digesting probiotic attenuates chronic colonic inflammation in spontaneous colitic mice model of IBD

Authors: Tanvi Shinde[1,2*] , Ravichandra Vemuri[2,3], Sonia Shastri[2], Agampodi Promoda Perera[2], Shakuntla V. Gondalia[4,5], David J. Beale[6], Avinash V. Karpe[6], Rajaraman Eri[2], Roger Stanley[1*]

[1]Centre for Food Innovation, Tasmanian Institute of Agriculture, University of Tasmania, Launceston, Tasmania 7250 Australia.

[2]Gut Health Research Group, School of Health Sciences, College of Health and Medicine, University of Tasmania, Launceston, Tasmania 7250 Australia.

[3]Department of Pathology, Wake Forest School of Medicine, Medical Center Boulevard, Winston-Salem NC 27157, USA.

[4]Centre for Human Psychopharmacology, Swinburne University of Technology, Hawthorn, Victoria, Australia.

[5] Health and Biosecurity, Commonwealth Scientific and Industrial Research Organization (CSIRO), Gate 13 Kintore Avenue 5000, South Australia, Australia.

[6]Land and Water, Commonwealth Scientific and Industrial Research Organization (CSIRO), Ecosciences Precinct, Dutton Park 4102, Queensland, Australia.

***Correspondence**: Tanvi Shinde

Email: tanvi.shinde@utas.edu.au

Phone: +61 4 79107345

Roger Stanley

Email: roger.stanley@utas.edu.au

Phone: +61 4 34565167

Keywords: Synbiotic, Prebiotic, Probiotic, IBD, *Bacillus* spores, whole plant sugarcane fibre, SCFAs, dysbiosis, gut microbiota

A.

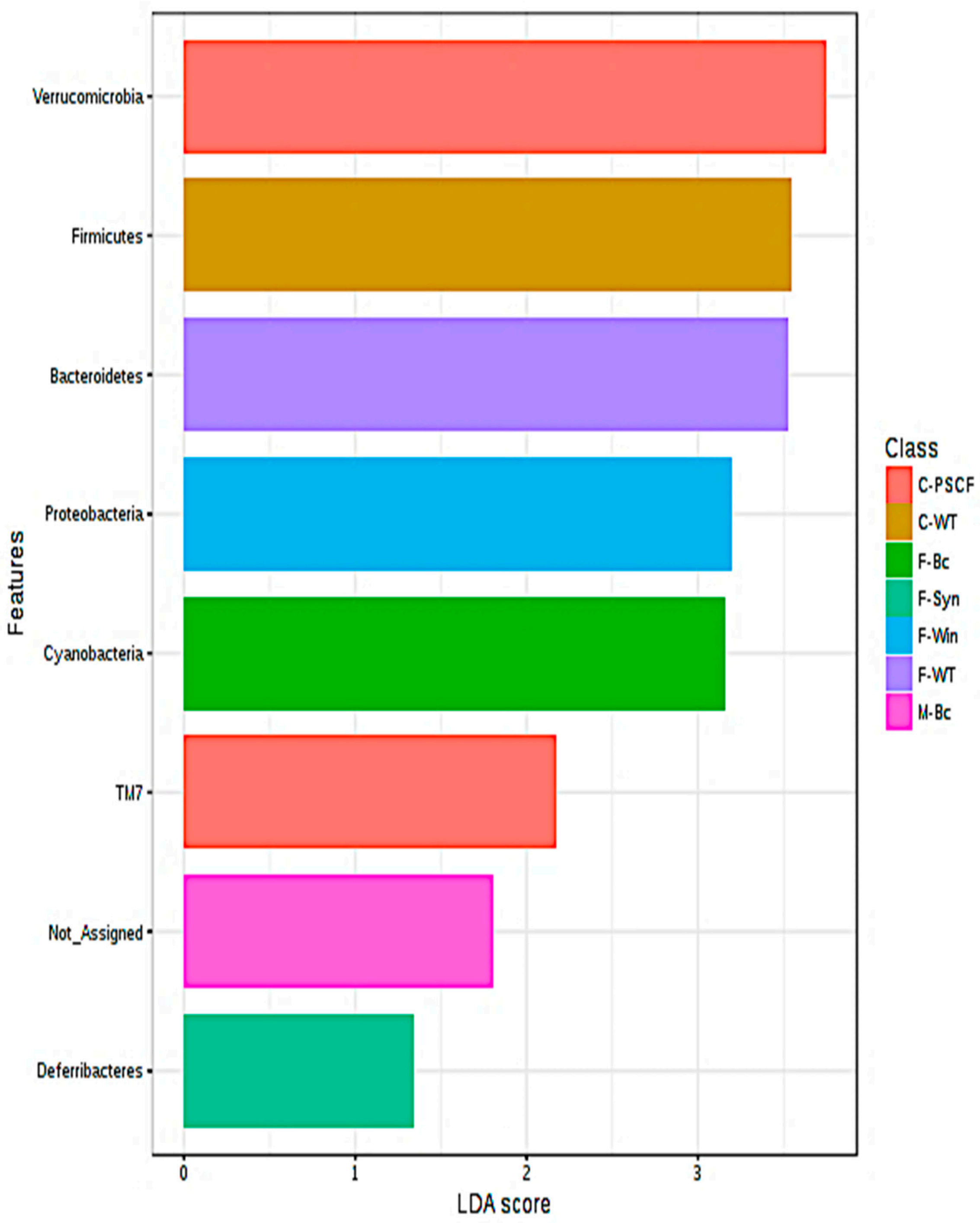
Verrucomicrobia
Firmicutes
Bacteroidetes
Proteobacteria
Cyanobacteria
TM7
Not_Assigned
Deferribacteres
Features
0
1
2
3
LDA score
Class
C-PSCF
C-WT
F-Bc
F-Syn
F-Win
F-WT
M-Bc

B.

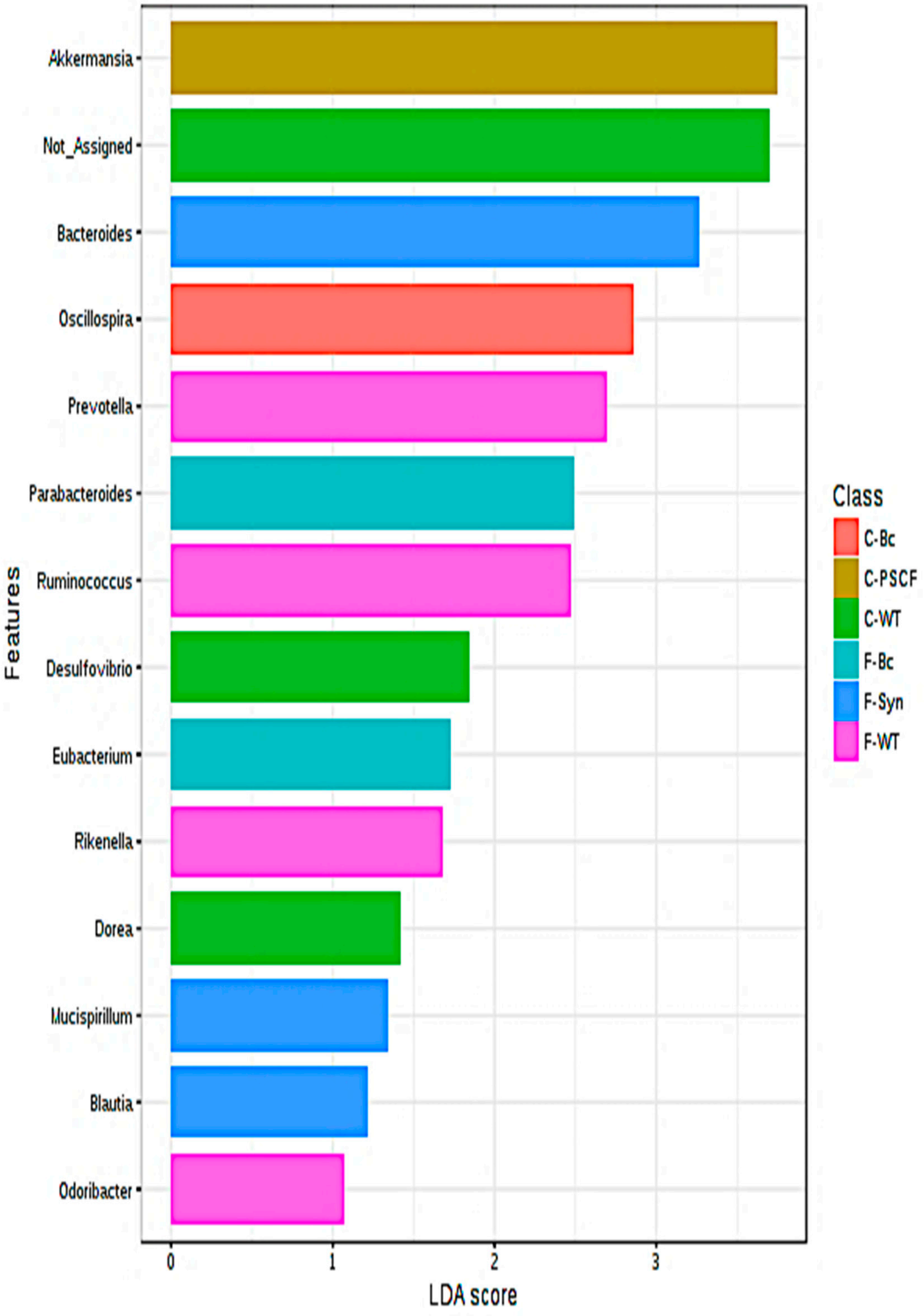

Akkermansia
Not_Assigned
Bacteroides
Oscillospira
Prevotella
Parabacteroides
Ruminococcus
Desulfovibrio
Eubacterium
Rikenella
Dorea
Mucispirillum
Blautia
Odoribacter
Features
0
1
2
3
LDA score
Class
C-Bc
C-PSCF
C-WT
F-Bc
F-Syn
F-WT

C.

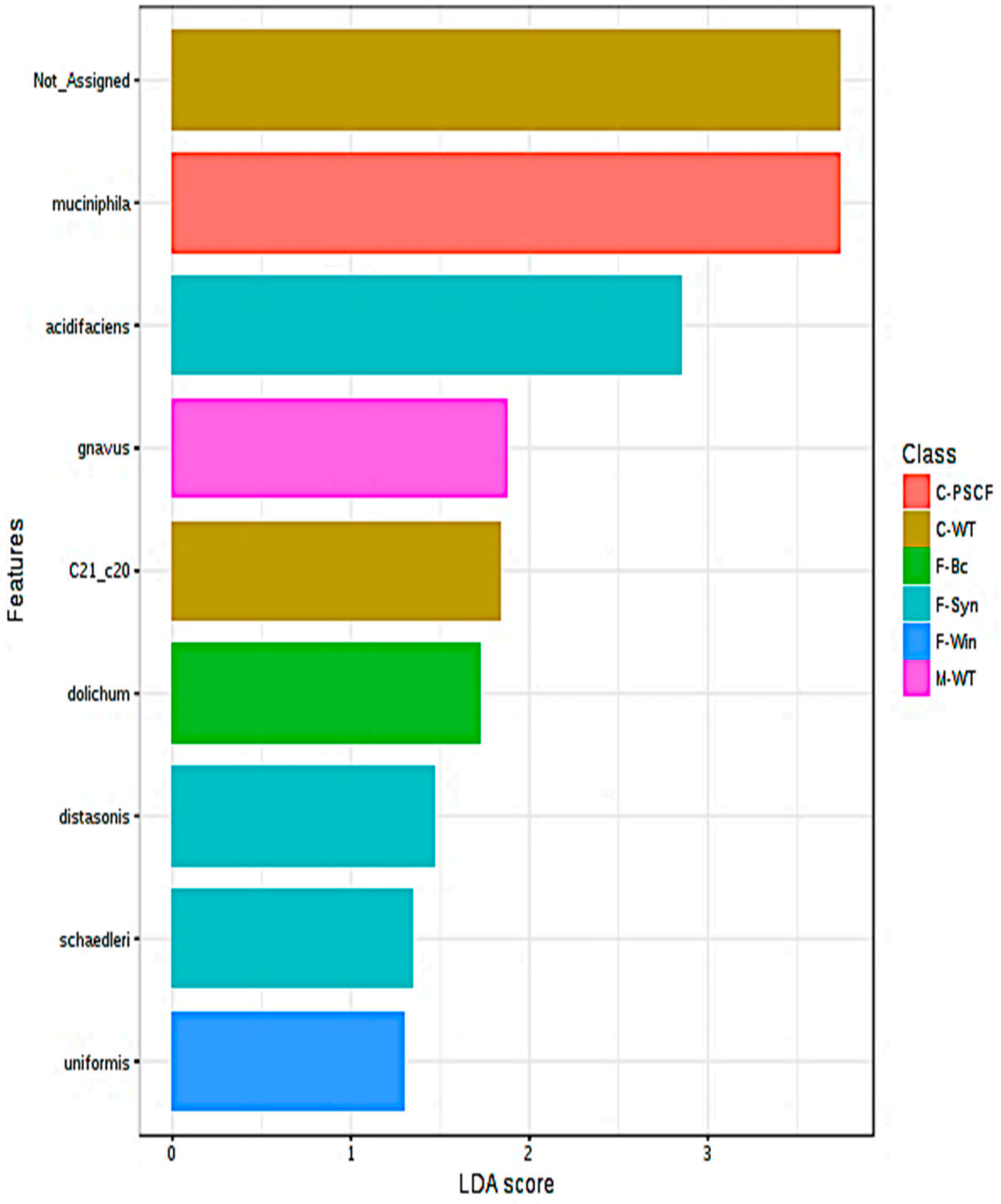

Figure S1. Biomarker analysis with Linear Discriminant Analysis (LDA) Effect Size (LEfSe) scoring plot using Kruskal-Wallis rank sum test (p = 0.01 and log LDA threshold cut-off value = 1.0)**.** Wild-type (WT), Winnie-control (Win), *B. coagulans* (Bc) spores, PSCF and synbiotic (Syn) groups at phylum (**A**), genus (**B**) and species (**C**) level. C-caecal, M-mucosal-associated, F-faecal.

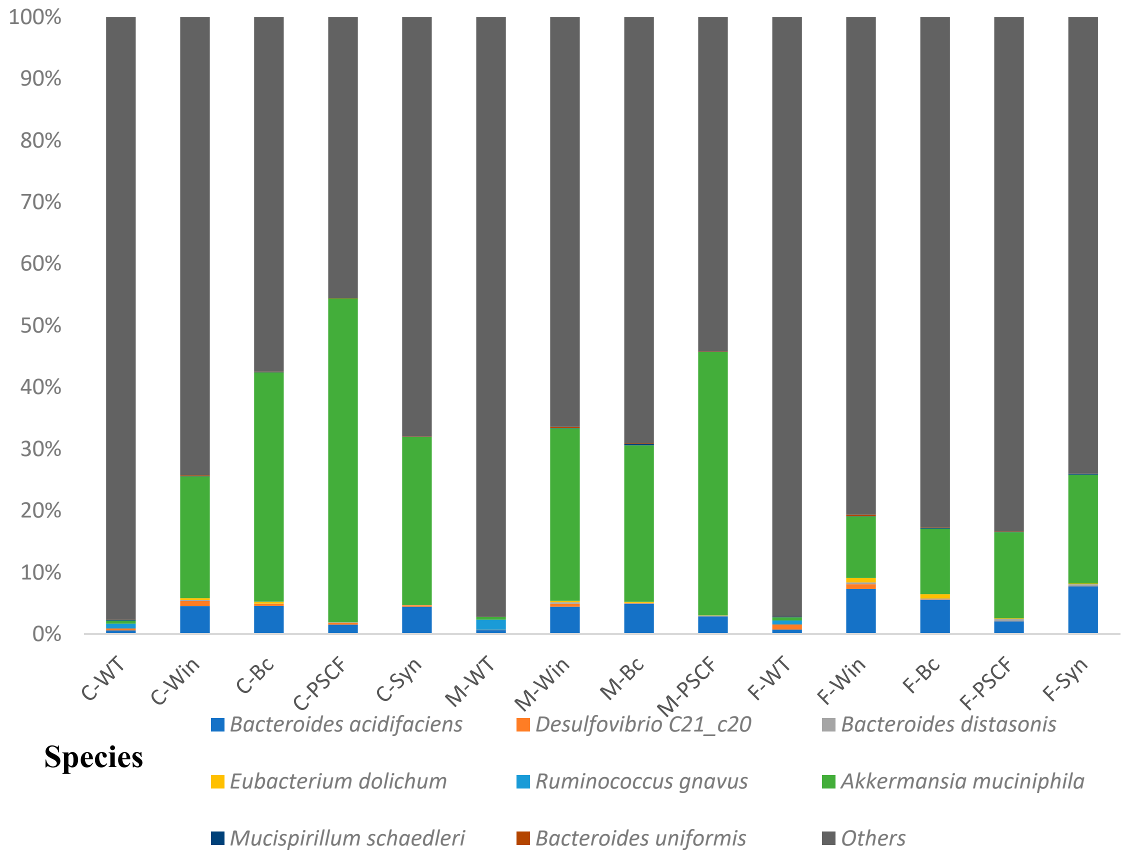

Figure S2. Relative abundances (%) of caecal (C)-, mucosal (M)- and Faecal (F)- associated microbiota at species level. Wild-type (WT), Winnie-control (Win), *B. coagulans* (Bc) spores, PSCF and Synbiotic (Syn) groups; (n = 5 per group).

Table S1. Nutritional information of Kfibre™ Prebiotic Sugarcane Fibre

Nutrient information of of Kfibre™ Prebiotic Sugarcane Fibre[a] (quantity per 100gm)	
Energy	950 kj
Protein	1.9 g
Total fat	3.1 g
-Saturated	1 g
Carbohydrates	92.5 g
-Sugars	8.1 g
Dietary fibre	84.5 g
Sodium	30 mg

[a]Ingredients: Sugarcane (sucrose reduced).
Information sourced from www.kfibre.com

Table S2. Nutritional information of mice standard chow diet

Nutrient information of standard rodent chow diet - Barastoc Mice Cubes[a]			
Minimal crude protein	20 %	Vitamin A	15 IU/g
Minimal crude fat	6 %	Vitamin D3	2 IU/g
Crude fibre	3.2 %	Vitamin E	260 mg/kg
Acid detergent fibre	4.4 %	Vitamin K3	55 mg/kg
Neutral detergent fibre	10.4 %	Vitamin B1	64 mg/kg
Digestible energy	12.8 MJ/kg	Vitamin B2	48 mg/kg
Calcium	1.14 %	Vitamin B6	30 mg/kg
Phosphorus	0.94 %	Vitamin B12	0.08 mg/kg
Sodium	0.35 %	Niacin	400 mg/kg
Potassium	0.82 %	Panto	220 mg/kg
Chloride	0.58 %	Biotin	1.48 mg/kg
Magnesium	0.24 %	Folic	11 mg/kg
Lysine	1.11 %	Iron	51 mg/kg
Methionine	0.37 %	Zinc	60 mg/kg
Linoleic	1.52 %	Manganese	120 mg/kg
Starch	29 %	Copper	10 mg/kg
Vitamin A	15 IU/g	Selenium	0.1 mg/kg
Vitamin D3	2 IU/g	Molybdenum	0.4 mg/kg
Vitamin E	260 mg/kg	Cobalt	0.6 mg/kg
Vitamin K3	55 mg/kg	Iodine	1 .4 mg/kg

[a] Ingredients include: wheat, wheat byproducts, groats (dehulled oats), meat meal, canola oil, soyabean meal, skim milk powder, molasses, salt, vitamins, trace minerals. The product is a complete and balanced diet to support the growth and health of mice and rats in laboratory environment.
Information sourced from www.ridley.com.au Product code 102108
